# The role of geriatric nutritional risk index in predicting survival of type B aortic dissection patients after thoracic endovascular aortic repair

**DOI:** 10.1016/j.jnha.2025.100572

**Published:** 2025-05-14

**Authors:** Kaiwen Zhao, Jinzhu Niu, Yuzhen He, Lingxu Kong, Wenyao Zhao, Qingsheng Lu, Shuangshuang Li, Jian Zhou

**Affiliations:** a Department of Vascular Surgery, Changhai Hospital of the Navy Medical University, Shanghai, China; b Department of General Surgery, The First Medical Center of the Chinese PLA General Hospital, Beijing, China; c Department of Vascular Surgery, The Third Affiliated Hospital of the Navy Medical University, Shanghai, China; d College of Clinical Medicine, Jining Medical University, Jining, Shandong, China

**Keywords:** Type B aortic dissection, Thoracic endovascular aortic repair, Malnutrition, GNRI

## Abstract

**Background:**

The geriatric nutritional risk index (GNRI) is a reliable indicator of patients’ nutrition status and has been shown to be valuable in predicting the outcome of patients with various cardiovascular diseases. This study explored the association between perioperative GNRI and the prognosis of type B aortic dissection (TBAD) patients receiving thoracic endovascular aortic repair (TEVAR).

**Methods:**

A total of 1,157 consecutive patients who underwent TEVAR between January 2007 and August 2019 were included, with data from 789 patients analyzed. The GNRI was used to measure nutritional status. Patients were categorized into five groups based on the GNRI quintile. The study's endpoints included all-cause mortality, aortic-related adverse events (ARAEs), and major adverse cardiovascular and cerebrovascular events (MACCEs) at 30 days, 1 year, and 5 years. The univariate and multivariate Cox regression analyses the effect of GNRI on the endpoints. Kaplan–Meier survival analysis was conducted to assess the incidence of these endpoints across the five groups, and restricted cubic spline (RCS) analysis was used to examine the non-linear relationship between GNRI and all-cause mortality.

**Results:**

The Kaplan-Meier survival analyses revealed that the risk of 1-year and 5-year all-cause mortality was highest in the Q1 group among the five groups (P = 0.009 and P = 0.002, respectively). However, there was no significant difference in 1-year and 5-year ARAEs and MACCEs (all P > 0.05). Multivariate Cox analysis showed that continuous GNRI was independently associated with 5-year all-cause death (HR = 0.97, 95% CI: 0.95–1.00; P = 0.027). Compared with the Q1 group, the Q2 (HR = 0.22, 95% CI: 0.06−0.80; P = 0.021) and Q4 groups (HR = 0.26, 95% CI: 0.08−0.81; P = 0.020) had lower risks of 1-year all-cause mortality. The Q2 group (HR = 0.38, 95% CI: 0.18−0.83; P = 0.015) and Q3 group (HR = 0.46, 95% CI: 0.22−0.96; P = 0.039) were also observed to have a lower risk of 5-year all-cause mortality than the Q1 group. In the subgroup analyses, chronic kidney disease (CKD) showed significant interaction (P-interaction < 0.001). Besides, the RCS analysis identified a “U”-shaped relationship between GNRI and all-cause mortality of TBAD patients following TEAVR.

**Conclusions:**

TBAD patients undergoing TEVAR showed a strong correlation between perioperative low GNRI and higher risks of 1-year and 5-year all-cause mortalities. TBAD patients with a too low GNRI should receive particular attention.

## 1 Introduction

Aortic dissection (AD) is one of the most devastating cardiovascular diseases (CVD), with high mortality, poor prognosis, and limited therapeutic options [1,2]. AD can be further classified based on the location of the dissection into Stanford type A aortic dissection (TAAD) and Stanford type B aortic dissection (TBAD). TBAD typically originates distal to the left subclavian artery (LSA), while the remaining cases are categorized as TAAD [3]. The treatment for TBAD patients mainly includes open surgical intervention, medical management, and thoracic aortic endovascular repair (TEVAR) [4]. TEVAR as an interventional therapy has demonstrated multiple advantages over traditional open surgery, including less trauma, favorable aortic remodeling, and faster postoperative recovery [3], which has been applied in growing numbers of TBAD patients [5]. Notwithstanding these advantages, the risk of long-term postoperative mortality persists, which is closely linked to retrograde type A dissection (RTAD), stroke, and multiple organ failures [[6], [7], [8]]. Therefore, it is imperative to identify diagnostically valuable markers and construct precious prognostic models for patient risk stratification, allowing for early intervention to prevent adverse events. With the increase in living standards, research into the relationship between nutrition status and the prognosis of CVD is receiving significant attention.

Malnutrition has been a serious health problem, especially in patients combined with CVD, and has raised a huge economic burden worldwide [9]. Both undernutrition and overweight are closely linked to alterations in circulating albumin, inflammatory cytokine [[10], [11], [12], [13]], which may contribute to the progression of atherosclerosis, arterial calcification, etc., and compromise patients’ life expectancy [14,15]. Although often underrecognized, malnutrition is also prevalent among individuals with aortic disorders and is correlated with poorer clinical outcomes [16]. For instance, AD patients frequently experience malnutrition as a consequence of a detrimental cycle characterized by increased absorption of endotoxins, diminished metabolic supplies, and a reduced clearance rate [17,18]. This cycle may ultimately lead to the development of aortic dilation and nutritional intervention has the potential to enhance both prognosis and quality of life in these patients. The Geriatric Nutritional Risk Index (GNRI) brought by Bouillanne et al. is a widely utilized and accessible metric for evaluating nutritional status [19]. It has been utilized in prognostic predictions for cardiovascular diseases, including ischemic heart failure, coronary artery disease, and chronic limb-threatening ischemia [[19], [20], [21]].

Currently, little emphasis has been devoted to the significance of GNRI in the prognosis of TBAD patients undergoing TEVAR. Thus, this study aimed to examine the association between perioperative GNRI and long-term outcomes of TBAD patients following TEVAR.

## 2 Methods

### 2.1 Study cohort and design

**Fig. 1:**
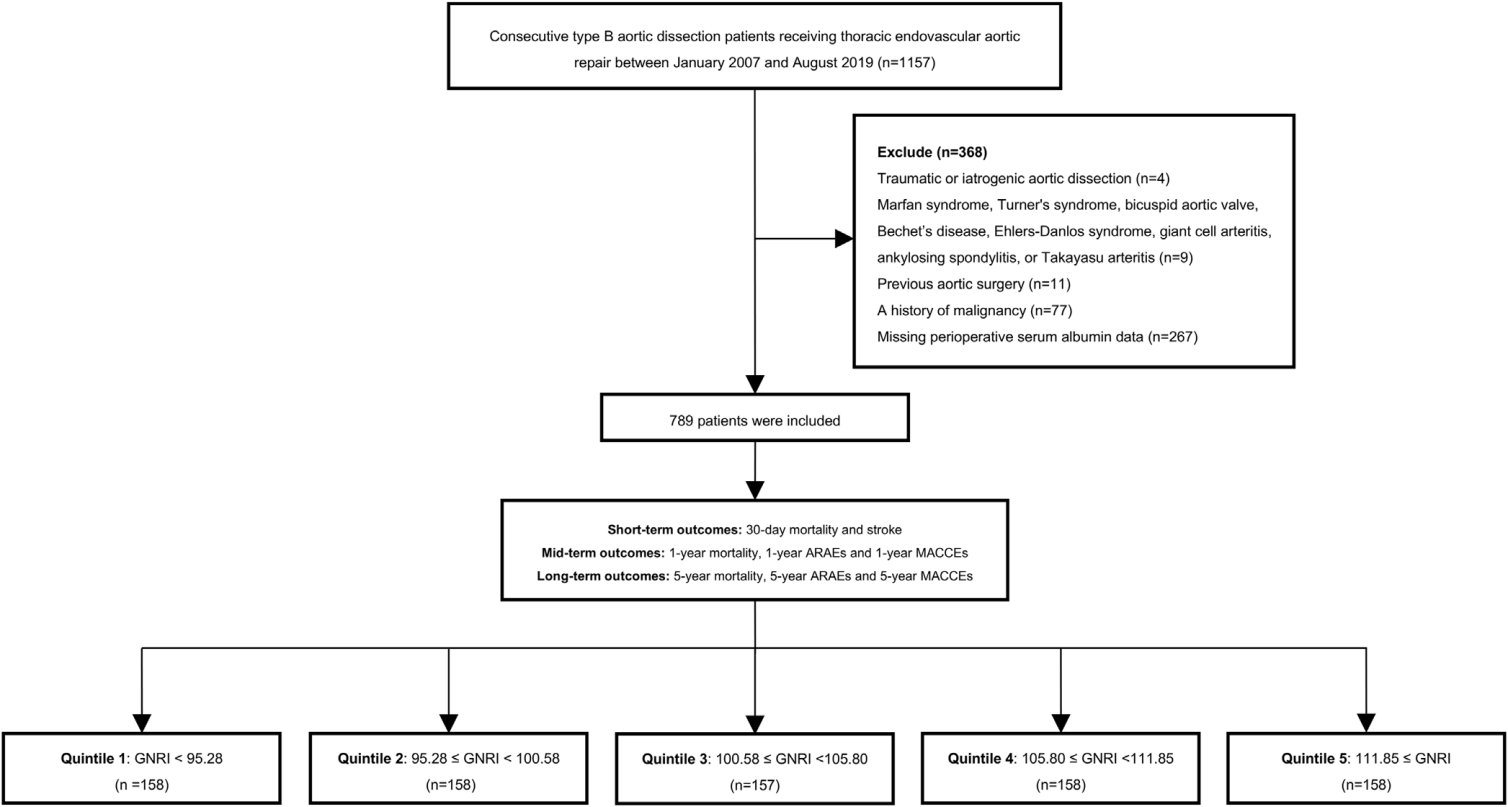
Flowchart of the patient selection process. MACCE, major adverse cardiovascular and cerebrovascular event; GNRI, geriatric nutritional risk index; ARAEs, aortic-related adverse events.

This study was a single-center, retrospective cohort study that enrolled 1,157 TBAD patients undergoing TEVAR in Shanghai Changhai Hospital from January 2007 to August 2019. The following patients were excluded: [1] instances of traumatic aortic injury as well as iatrogenic aortic dissection (n = 4) [2]; the presence of Turner’s syndrome, Marfan syndrome, Ehlers-Danlos syndrome, bicuspid aortic valve, giant cell arteritis, ankylosing spondylitis, Bechet’s disease, or Takayasu arteritis (n = 9) [3]; a history of prior aortic surgical intervention (n = 11) [4]; a documented history of malignancy (n = 77); and [5] absence of perioperative serum albumin data (n = 267). Following the above exclusions, a total of 789 patients were included in this study (Fig. 1). The research protocol received approval from the central ethics committee of the Shanghai Changhai Hospital (CHEC-Y2020-042). Given the retrospective design of the study, the requirement for consent forms was waived.

### 2.2 Data collection and definitions

Presentations of TBAD were divided into the following three groups based on the duration of clinical onset: acute (less than or equal to 14 days), subacute (15–90 days), and chronic (greater than or equal to 91 days) [22]. Aortic-related adverse events (ARAEs) encompass a range of complications, including RTAD, aortic rupture, aortic dilation, malperfusion, and type I or III endoleak [23]. Chronic kidney disease (CKD) was characterized by an estimated GRF (eGFR) of less than 60 mL/min/1.73 m^2^ [24]. Demographics, smoking status, comorbidities, image information, medical history, laboratory parameters, and intraoperative details were derived from the record system of the Shanghai Changhai Hospital. D-dimer, serum albumin, creatinine, hemoglobin, glucose, total cholesterol, low-density lipoprotein cholesterol (LDL-c), triglycerides (TG), and high-density lipoprotein cholesterol (HDL-c) were assessed utilizing standard biochemical methodologies at the Shanghai Changhai Hospital Clinical Laboratory. For surgical procedures that were not conducted in an urgent or emergency context, blood samples and body weight data were collected on the morning prior to the surgery while the patients were in a fasting state. For emergency surgeries, these measurements were collected in the emergency room or during surgery. The calculation of the GNRI is performed utilizing the subsequent formula: [1.489×albumin(g/L)]+[41.7×(ratio of dry weight to ideal weight)] [25,26]. According to GNRI quintiles, the participants were divided into five distinct groups [Q1 (n = 158, GNRI < 95.28), Q2 (n = 158, 95.28 ≤ GNRI < 100.58), Q3 (n = 157, 100.58 ≤ GNRI < 105.80), Q4 (n = 158, 105.80 ≤ GNRI < 111.85), and Q5 (n = 158, 111.85 ≤ GNRI)].

### 2.3 Follow-up and endpoints

The objectives of the study encompassed various outcomes categorized by timeframes: short-term outcomes, which included all-cause mortality and stroke within a 30-day period; mid-term outcomes, comprising all-cause mortality, ARAEs, and major adverse cardiovascular and cerebrovascular events (MACCEs) at the 1-year mark; and long-term outcomes consisting of 5-year mortality, 5-year ARAEs, and 5-year MACCEs. When adverse events happened more than once, only the initial instance was analyzed. The study was carried out by credentialed researchers utilizing medical records, telephone interviews, and surveys. Additionally, adverse events were assessed through a thorough review of clinical records that necessitated re-admission or were assessed during clinic visits. The adverse events and endpoints were assessed by two independent physicians with expertise in the diagnosis and management of TBAD, who were blinded to the clinical information of the patients.

### 2.4 Statistical analysis

In this study, the mean and standard deviation (SD) were used as the normally distributed continuous variables. Cumulative survival curves were estimated using Kaplan-Meier (KM) methodologies, and comparisons between various groups were performed using the log-rank tests. Expressing categorical variables as percentages and analyzing using chi-square or Fisher exact tests. The research utilized Cox proportional hazards regression analysis to estimate the hazards ratio (HR) and 95% confidence interval (CI) for the study endpoints. The relationship between the preoperative GNRI and all-cause mortality outcomes at both 1 year and 5 years was analyzed using univariate and multivariate Cox regression models. Variables exhibiting a significance level of P < 0.01 in the univariate analysis were incorporated into the multivariate Cox proportional hazard models. In order to compare variables with continuous distributions, a Mann-Whitney test or a student t-test utilizing medians, means, or standard deviations (from the Q1 group to the Q5 group) was employed. Initially, the GNRI was provided as a continuous variable before it was subsequently categorized for modeling purposes. Furthermore, potential nonlinear associations between continuous variables and their respective outcomes were evaluated using generalized additive models (GAM) incorporating restricted cubic splines (RCS) with four degrees of freedom. Statistical analyses were performed utilizing R software, version 3.6.3, in conjunction with EmpowerStats software (available at http://www.empowerstats.com). A significance level of P < 0.05 was established as the threshold for statistical significance.

## 3 Results

### 3.1 Clinical characteristics

Table 1 presents the baseline characteristics of the patients diagnosed with TBAD. The mean age of the 789 patients included in the study was 59.5 ± 13.5 years. Among the participants, 639 were male, constituting 81.0% of the population. In all analyzed groups, the Q1 group exhibited a significantly higher age compared to the four groups (Q1 vs. Q2 vs. Q3 vs. Q4 vs. Q5 = 63.2 ± 14.8 vs. 61.5 ± 13.4 vs. 58.7 ± 13.3 vs. 59.5 ± 12.3 vs. 54.6 ± 12.4, P < 0.001). The Q3 group had the highest percentage of males, and the Q1 group had the lowest among the five groups (P = 0.083). The BMI differed significantly among the five groups, with the Q5 group having a BMI that was noticeably greater than the other four groups (Q1 vs. Q2 vs. Q3 vs. Q4 vs. Q5 = 21.1 ± 2.8 vs. 22.9 ± 2.0 vs. 24.0 ± 2.3 vs. 25.6 ± 2.7 vs. 28.3 ± 3.3, P < 0.001). The systolic and diastolic blood pressures recorded upon admission did not exhibit statistically significant differences across the groups (all P > 0.05). No statistically significant differences were observed in the prevalence of the following comorbidities, including coronary artery disease (CAD), stroke, systolic blood pressure (SBP), diastolic blood pressure (DBP), and chronic obstructive pulmonary disease (COPD) across the various groups (all P > 0.05). The distribution of hypertension (HBP), diabetes mellitus, and CKD differed significantly among the five groups (P = 0.015, 0.004 and P < 0.001, respectively). The concentration of hemoglobin, triglycerides, and glucose was found to be significantly higher, while creatinine was significantly lower in the Q5 group compared with the other four groups (P < 0.001, P < 0.001, P = 0.001, and P < 0.001, respectively). There was no significant difference in the other laboratory parameters, which included white blood cell count (WBC), platelet count, total cholesterol, LDL-c, and HDL-c (all P > 0.05). There were no statistically significant differences in the application of branch, adjunct, and hybrid methodologies across groups demonstrates (all P > 0.05). The Q1 group and Q3 group had significantly fewer patients in the acute phase, and the Q1 group exhibited a higher number of patients in the sub-acute phase compared to the other groups (P = 0.047).

**Table 1:** Baseline characteristics of TBAD patients grouped according to quintile of the GNRI.

Variable	GNRI						*P*-value
	Total	Quintile 1	Quintile 2	Quintile 3	Quintile 4	Quintile 5	
N	789	158	158	157	158	158	
Age (years)	59.5 ± 13.5	63.2 ± 14.8	61.5 ± 13.4	58.7 ± 13.3	59.5 ± 12.3	54.6 ± 12.4	<0.001
Male	639 (81.0)	122 (77.2)	121 (76.6)	131 (83.4)	138 (87.3)	127 (80.4)	0.083
BMI, kg/m²	24.4 ± 3.6	21.1 ± 2.8	22.9 ± 2.0	24.0 ± 2.3	25.6 ± 2.7	28.3 ± 3.3	<0.001
Smoking	432 (54.8)	97 (61.4)	97 (61.4)	86 (54.8)	77 (48.7)	75 (47.5)	0.023
SBP at admission, mmHg	137.1 ± 20.8	135.7 ± 22.3	137.7 ± 20.9	136.1 ± 19.1	135.4 ± 17.8	140.6 ± 23.3	0.146
DBP at admission, mmHg	82.3 ± 11.2	81.6 ± 13.3	82.2 ± 11.2	81.5 ± 8.8	81.6 ± 8.9	84.5 ± 12.8	0.069
**Comorbidities**							
Hypertension	590 (74.8)	105 (66.5)	118 (74.7)	118 (75.2)	117 (74.1)	132 (83.5)	0.015
Diabetes mellitus	70 (8.9)	9 (5.7)	9 (5.7)	23 (14.6)	9 (5.7)	20 (12.7)	0.004
Stroke	46 (5.8)	9 (5.7)	7 (4.4)	13 (8.3)	10 (6.3)	7 (4.4)	0.571
CAD	46 (5.8)	10 (6.3)	7 (4.4)	13 (8.3)	11 (7.0)	5 (3.2)	0.312
COPD	94 (11.9)	25 (15.8)	24 (15.2)	13 (8.3)	19 (12.0)	13 (8.2)	0.094
CKD	50 (6.3)	25 (15.8)	8 (5.1)	6 (3.8)	8 (5.1)	3 (1.9)	<0.001
Pericardial effusion	55 (7.0)	17 (10.8)	10 (6.3)	11 (7.0)	10 (6.3)	7 (4.4)	0.261
Pleural effusion	280 (35.5)	67 (42.4)	64 (40.5)	50 (31.8)	50 (31.6)	49 (31.0)	0.08
**Laboratory tests**							
WBC, ×10^9^/L	8.7 ± 3.7	8.4 ± 3.5	8.5 ± 4.1	8.4 ± 3.2	9.1 ± 4.1	9.1 ± 3.5	0.203
Platelet, ×10^9^/L	205.9 ± 81.7	214.7 ± 102.4	207.1 ± 84.9	206.2 ± 71.2	196.9 ± 76.4	204.3 ± 67.4	0.444
Hemoglobin, g/L	128.9 ± 19.5	113.4 ± 20.3	125.7 ± 17.4	131.5 ± 16.4	133.5 ± 13.8	140.7 ± 17.5	<0.001
Creatinine, μmol/L	103.5 ± 115.3	143.8 ± 186.7	93.1 ± 99.1	110.6 ± 131.2	88.7 ± 39.7	81.1 ± 27.9	<0.001
Glucose, mg/dl	6.6 ± 2.5	6.6 ± 4.0	6.2 ± 1.8	6.5 ± 2.0	6.6 ± 1.8	6.9 ± 2.1	0.001
Total cholesterol, mg/dL	171.4 ± 40.5	166.7 ± 41.5	167.2 ± 38.9	171.5 ± 35.3	174.8 ± 45.0	176.9 ± 40.8	0.186
Triglycerides, mg/dL	124.4 ± 88.4	98.9 ± 46.5	114.5 ± 63.7	132.9 ± 100.2	134.0 ± 83.4	142.2 ± 123.3	<0.001
LDL-c, mg/dL	46.8 ± 126.6	41.7 ± 81.3	51.6 ± 186.5	44.9 ± 48.8	34.4 ± 41.8	62.7 ± 189.0	0.486
HDL-c, mg/dL	45.2 ± 13.3	45.4 ± 16.2	46.5 ± 14.4	45.0 ± 12.5	45.5 ± 11.7	43.6 ± 11.1	0.567
**Intra-operative details**							
Timing of operation							0.047
Acute	472 (59.8)	84 (53.2)	101 (63.9)	87 (55.4)	105 (66.5)	95 (60.1)	
Sub-acute	231 (29.3)	62 (39.2)	41 (25.9)	46 (29.3)	37 (23.4)	45 (28.5)	
Chronic	86 (10.9)	12 (7.6)	16 (10.1)	24 (15.3)	16 (10.1)	18 (11.4)	
Branch	144 (18.3)	23 (14.6)	30 (19.0)	30 (19.1)	24 (15.2)	37 (23.4)	0.249
Adjunct	127 (16.1)	27 (17.1)	31 (19.6)	24 (15.3)	21 (13.3)	24 (15.2)	0.616
Hybrid	12 (1.5)	1 (0.6)	3 (1.9)	1 (0.6)	3 (1.9)	4 (2.5)	0.553
**Anatomical characteristics**							
Dissection length	414.7 ± 144.5	373.2 ± 155.4	418.6 ± 154.4	358.4 ± 146.6	432.3 ± 157.9	454.5 ± 116.7	0.118
Proximal thrombosis of FL							0.085
Patent	339 (43.0)	66 (41.8)	59 (37.3)	73 (46.5)	65 (41.1)	75 (47.5)	
Partial	241 (30.5)	48 (30.4)	54 (34.1)	52 (33.1)	44 (27.8)	44 (27.8)	
Complete	122 (15.5)	16 (10.1)	29 (18.4)	19 (12.1)	31 (19.6)	25 (15.8)	
ULP	87 (11.0)	28 (17.7)	16 (10.1)	13 (8.3)	18 (11.4)	14 (8.8)	
Superior mesenteric arteries	1 (0.1)	0	1 (0.6)	0	0	0	0.406
Renal arteries	29 (3.7)	10 (6.3)	5 (3.2)	6 (3.8)	6 (3.8)	2 (1.3)	0.209
Lower-extremity arteries	7 (0.9)	1 (0.6)	3 (1.9)	0	2 (1.3)	1 (0.6)	0.443

Values are presented as n (%), mean ± SD. Categorical variables were presented as n (%). TBAD, type B aortic dissection; GNRI, geriatric nutritional risk index; BMI, body mass index; SBP, systolic blood pressure; DBP, diastolic blood pressure; COPD, chronic obstructive pulmonary disease; CKD, chronic kidney disease; CAD, coronary artery disease; WBC, white blood cell; LDL-C, low-density lipoprotein cholesterol; HDL-C, high-density lipoprotein cholesterol; FL, false lumen; ULP, ulcer-like projection.

### 3.2 30-day outcomes

The outcomes observed over a 30-day period are presented in Table 2. No statistical significance was observed in the average duration of hospital stay post-TEVAR across all groups (P = 0.891). During hospitalization or within the 30-day period following TEVAR, the mortality rates among the five study groups did not exhibit statistically significant differences (Q1 vs. Q2 vs. Q3 vs. Q4 vs. Q5 = 4.4% vs. 1.3% vs. 1.9% vs. 1.3% vs. 0.6%, P = 0.113). Furthermore, the study showed no statistically significant difference in the incidence of 30-day stroke following TEVAR (P = 0.404).

**Table 2:** Outcomes of TBAD patients receiving TEVAR grouped according to quintiles of the GNRI.

Variable	Quintile 1	Quintile 2	Quintile 3	Quintile 4	Quintile 5	*P*-value
Hospital stays of post-TEVAR, days	11.6 ± 5.6	11.8 ± 6.0	12.3 ± 6.4	11.8 ± 7.0	12.2 ± 6.4	0.891
30-day outcomes						
30-day mortality, n (%)	7 (4.4)	2 (1.3)	3 (1.9)	2 (1.3)	1 (0.6)	0.113
30-day stroke, n (%)	2 (1.3)	2 (1.3)	0	0	2 (1.3)	0.404
1-year outcomes						
Cumulative incidence of 1-year all-cause death, n (%)	10.69 (5.16−15.89)	2.25 (0−4.75)	6.37 (2.23−10.34)	2.90 (0.02−5.69)	4.49 (0.89−7.96)	0.009
Cumulative incidence of 1-year ARAEs, n (%)	11.42 (5.51−16.97)	7.43 (2.87−11.79)	14.99 (8.84−20.73)	9.66 (4.45−14.58)	13.2 (7.28−18.73)	0.379
Cumulative incidence of 1-year MACCEs, n (%)	4.07 (0.44−7.58)	3.00 (0.05−5.86)	3.14 (0.05−6.13)	0	4.39 (0.87−7.79)	0.222
5-year outcomes						
Cumulative incidence of 5-year all-cause death, n (%)	25.39 (14.96−34.54)	10.14 (3.21−16.58)	12.56 (3.75−20.56)	12.43 (4.33−19.84)	7.94 (2.67−12.94)	0.002
Cumulative incidence of 5-year ARAEs, n (%)	25.21 (14.60−34.49)	22.19 (11.1−31.89)	29.69 (18.66−39.23)	27.08 (15.34−37.19)	21.52 (12.31−29.75)	0.519
Cumulative incidence of 5-year MACCEs, n (%)	6.61 (1.57−11.39)	5.25 (0.96−9.36)	6.26 (1.56−10.74)	7.10 (0−13.74)	11.47 (3.39−18.88)	0.624

Values are presented as n (%), mean ± SD. Categorical variables were presented as n (%). TBAD, type B aortic dissection; TEVAR, thoracic endovascular aortic repair; ARAEs, aortic-related adverse events; GNRI, geriatric nutritional risk index; MACCEs, major adverse cardiovascular and cerebrovascular events.

### 3.3 1-year outcomes

**Fig. 2:**
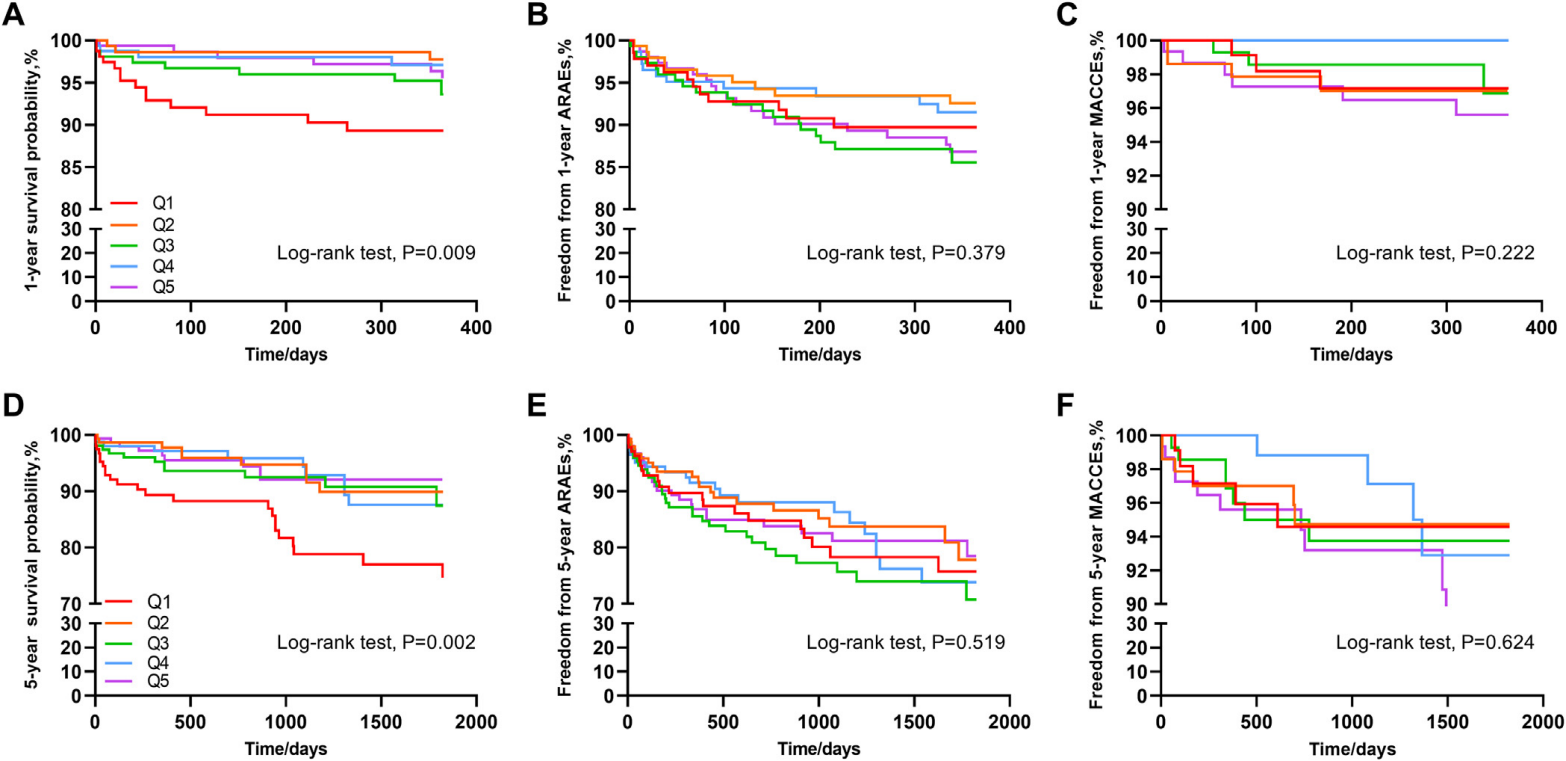
Kaplan-Meier survival analysis of 1-year and 5-year outcomes for TBAD patients undergoing TEVAR. A. The 1-year overall survival probability. B. The 1-year freedom from ARAEs. C. The 1-year freedom from MACCEs. D. The 5-year overall survival probability. E. The 5-year freedom from ARAEs. F. The 5-year freedom from MACCEs. TBAD, type B aortic dissection; TEVAR, thoracic endovascular aortic repair; MACCEs, major adverse cardiovascular and cerebrovascular events; ARAEs, aortic-related adverse events; Q1, quintile 1; Q2, quintile 2; Q3, quintile 3; Q4, quintile 4; Q5, quintile 5.

The cumulative incidence of ARAEs, MACCEs, and all-cause mortality during 1 year is shown in Table 2. A total of 76 ARAEs, 19 MACCEs, and 36 fatalities were found during the 1-year follow-up. It is worth mentioning that among the five study groups, there was the highest risk of 1-year all-cause mortality in the Q1 group (10.69%) and the lowest in the Q2 group (2.25%) (P = 0.009) **(**Table 2) (Fig. 2). Nonetheless, the analysis revealed no statistically significant variation in the cumulative incidence of both 1-year ARAEs and 1-year MACCEs across the five groups (P = 0.379 and P = 0.222, respectively) (Table 2).

**Fig. 3:**
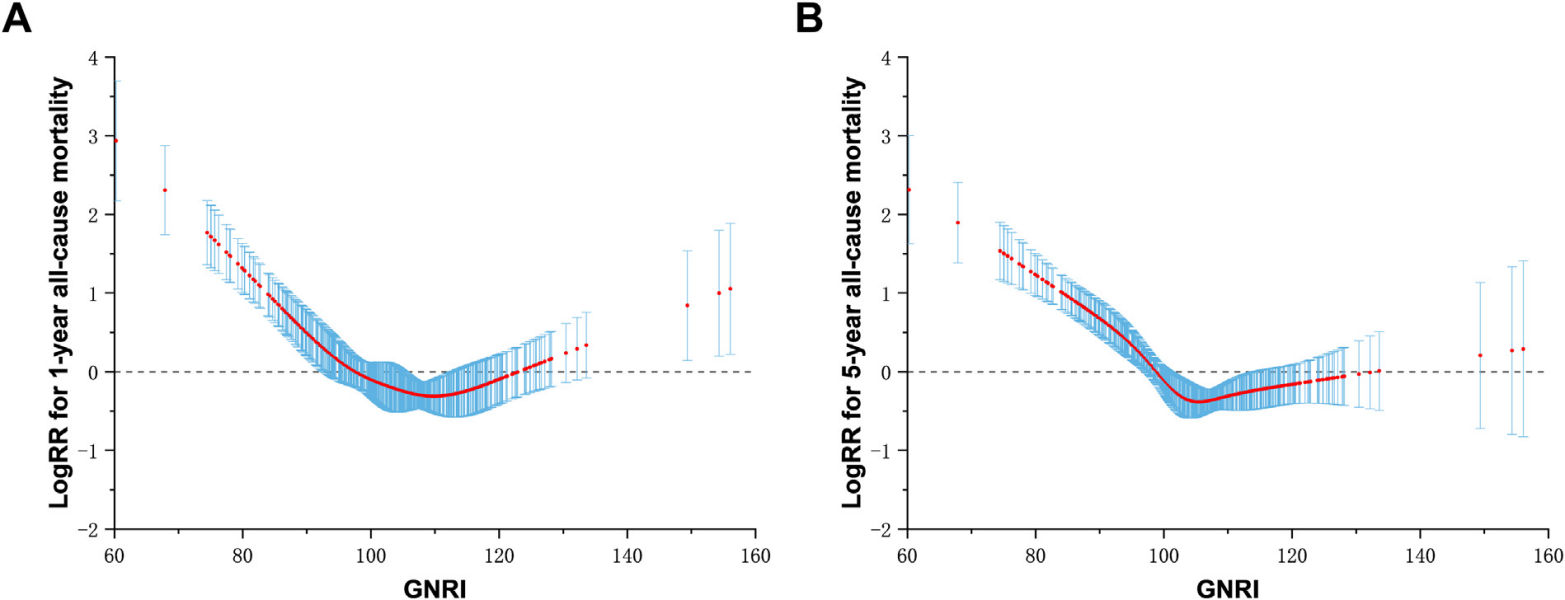
The association between GNRI and 1-year outcomes as well as 5-year outcomes. A. The relationship between GNRI and logRR for 1-year mortality. B. The relationship between GNRI and log RR for 5-year mortality. GNRI, geriatric nutritional risk index; RR, relative risk.

The results of the Cox proportional hazards model analysis are shown in Table 3. Univariate Cox proportional hazards analysis revealed that sex, diabetes mellitus, stroke, CKD, pericardial effusion, and GNRI (categorical) were significantly correlated with all-cause mortality at the 1-year mark (P < 0.05) (Supplementary Tables S1 and S2). Table 3 also illustrates a statistically significant relationship between GNRI (categorical) and 1-year all-cause mortality after controlling for variables such as stroke, CKD, and pericardial effusion (P < 0.1 in the univariate analysis). Both the Q2 (HR = 0.22, 95% CI: 0.06−0.80; P = 0.021), Q4 groups (HR = 0.26, 95% CI: 0.08−0.81; P = 0.020) exhibited a reduced risk of 1-year all-cause mortality compared to the Q1 group. Furthermore, this study demonstrates a U-shaped relationship between GNRI and the incidence of 1-year all-cause mortality, as evidenced by RCS (Fig. 3). When the GNRI fell below 98 or above 122, as illustrated in the curve, the logRR for 1-year all-cause mortality was markedly increased.

**Table 3:** Association between GNRI and mid- and long-term outcomes.

Variable	Univariate Cox regression model				Multivariate Cox regression model			
	1-year all-cause death	*P*-value	5-year all-cause death	*P*-value	1-year all-cause death	*P*-value	5-year all-cause death	*P*-value
Continuous GNRI	1.00 (0.99, 1.00)	0.022	0.99 (0.99, 1.00)	<0.001	0.97 (0.94, 1.01)	0.107	0.97 (0.95, 1.00)	0.027
GNRI groups								
Quintile 1	Reference		Reference		Reference		Reference	
Quintile 2	0.19 (0.05, 0.66)	0.009	0.33 (0.15, 0.71)	0.005	0.22 (0.06, 0.80)	0.021	0.38 (0.18, 0.83)	0.015
Quintile 3	0.55 (0.24, 1.27)	0.162	0.44 (0.22, 0.88)	0.020	0.59 (0.24, 1.48)	0.260	0.46 (0.22, 0.96)	0.039
Quintile 4	0.26 (0.08, 0.78)	0.017	0.38 (0.18, 0.79)	0.010	0.26 (0.08, 0.81)	0.020	0.47 (0.22, 1.00)	0.051
Quintile 5	0.36 (0.14, 0.94)	0.038	0.31 (0.14, 0.67)	0.003	0.50 (0.18, 1.40)	0.186	0.48 (0.22, 1.06)	0.070

Covariates for the multivariable model include age, gender, smoking, systolic blood pressure; diastolic blood pressure; hypertension, diabetes, stroke, chronic obstructive pulmonary disease; chronic kidney disease; coronary artery disease; timing of operation; pericardial effusion; pleura effusion, branch; adjunct; hybrid. Variables with a P value < 0.1 in univariable analysis were entered in the multivariable models (Details in the Supplementary Tables S1–S4). GNRI, Geriatric nutritional risk index; CI, confidence interval; HR, hazards ratio; OR, odds ratio; NA, not applicable.

### 3.4 5-year outcomes

In the 5-year outcomes analysis, the analysis revealed no statistically significant difference in the cumulative incidence of ARAE and MACCE among the five groups (P = 0.519 and P = 0.624, respectively) (Table 2). During the 5-year follow-up period, 64 fatalities, 122 ARAEs, and 34 MACCEs were observed. As a result, the Q1 group had the highest mortality rate and Q5 group had the lowest mortality rate out of the five groups (P = 0.002) (Table 2) (Fig. 2).

According to the results of univariate Cox proportional hazards analysis, age, diabetes, stroke, COPD, CKD, and pericardial effusion, GNRI (continuous and categorical) were significantly correlated with all-cause mortality over a 5-year period (P < 0.05) (Supplementary Tables S3 and S4). There was a statistically significant relationship between the GNRI (continuous) and 5-year all-cause death (HR = 0.97, 95% CI: 0.95–1.00, P = 0.027) by the result of multivariate Cox regression analysis. As summarized in Table 3, there was still a significant statistical relationship between GNRI (categorical) and 5-year all-cause mortality rate after adjusting for age, CAD, diabetes, stroke, COPD, CKD, and pericardial effusion. The Q2 group (HR = 0.38, 95% CI: 0.18−0.83; P = 0.015), Q3 group (HR = 0.46, 95% CI: 0.22−0.96; P = 0.039) were still observed to have a lower risk of 5-year all-cause mortality than the Q1 group.

**Fig. 4:**
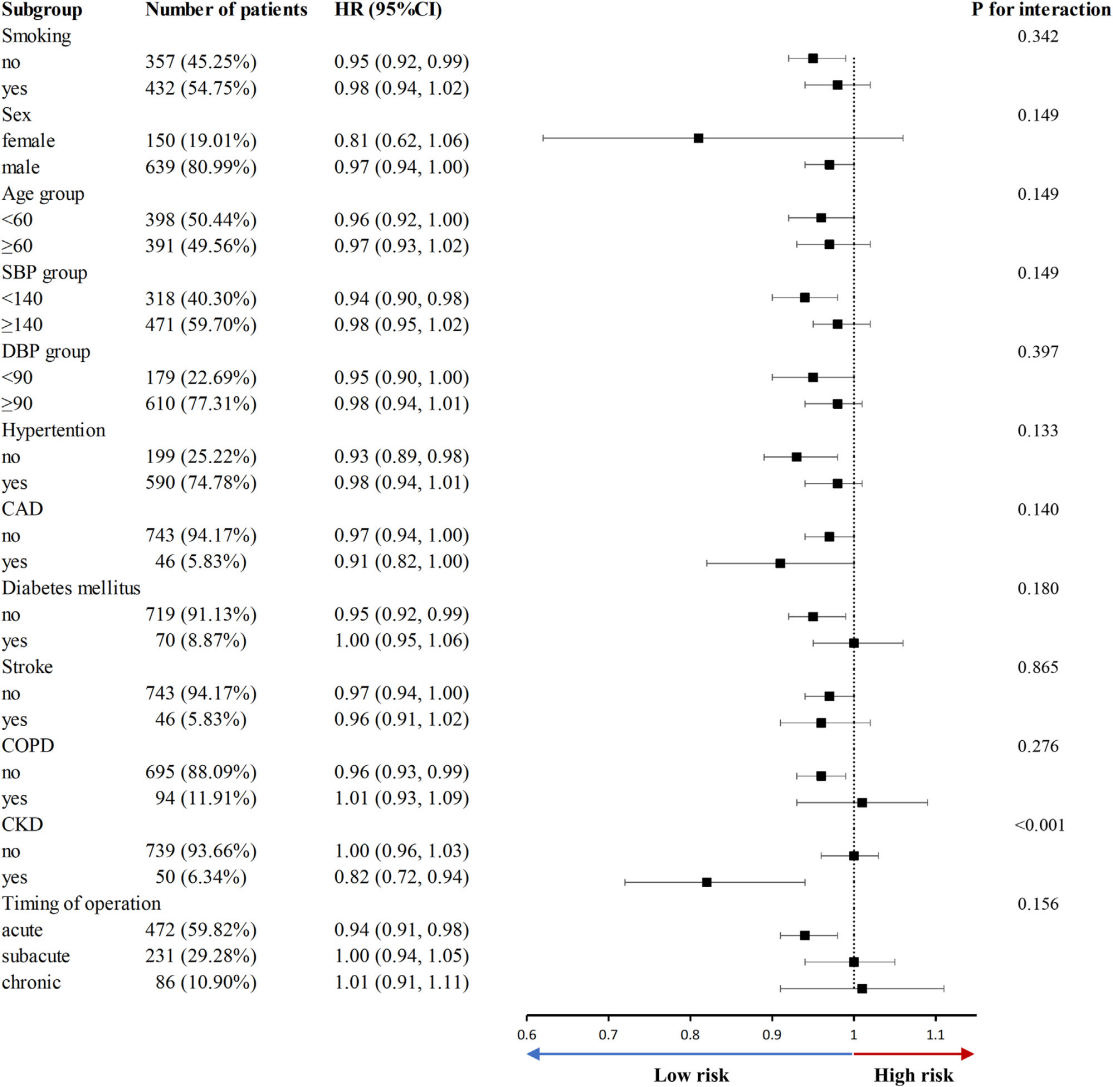
Subgroup analysis for association between GNRI and 5-year all-cause mortality. SBP, systolic blood pressure; DBP, diastolic blood pressure; COPD, chronic obstructive pulmonary disease; CKD, chronic kidney disease; CAD, coronary artery disease; HR, hazards ratio; CI, confidence interval.

Additionally, to evaluate the influence of various potential confounding variables on the outcomes, including CAD, stroke, diabetes mellitus, CKD, SBP, DBP, HBP, gender, age, smoking status, COPD, and surgical timing, we performed subgroup analyses (Fig. 4). Subgroup analyses indicated a significant interaction between CKD and GNRI (P-interaction < 0.001), demonstrating a pronounced negative correlation between GNRI and 5-year all-cause mortality within the CKD cohort, while not significant in the non-CKD population. Except for CKD, there was no observed interaction between the GNRI and any of the subgroups analyzed (all P-interaction > 0.05).

The RCS analysis identified a U-shaped relationship between GNRI and 5-year all-cause death (Fig. 3). Notably, the logRR for 5-year all-cause mortality was obviously elevated in individuals with GNRI values both below 100 and above 130.

## 4 Discussion

The current study demonstrates that TBAD patients with lower GNRI experienced a higher risk of all-cause mortality in mid-term and long-term outcomes. Even after adjusting for potential confounders, GNRI remained independently associated with higher mortality risk. RCS analysis revealed a “U” shaped relationship between GNRI and all-cause mortality. These results imply that GNRI is a reliable index for predicting TBAD patients’ mid-term and long-term risk when receiving TEVAR therapy.

Preoperative nutritional support plays a critical role for the surgical patient that reduces metabolic stress and post-surgery recovery [27]. Research has proven that malnutrition, particularly in patients with CVD, may lead to adverse outcomes following surgery [28]. Therefore, it is essential to conduct a precise evaluation of a patient's nutritional status and implement an appropriate nutritional intervention accordingly. However, conventional assessment criteria, such as the mini nutritional assessment (MNA), subjective global assessment (SGA), and controlling nutritional status (CONUT), are relatively complex and insufficient to meet the clinical needs for evaluating patients' nutritional status [[29], [30], [31]]. The GNRI is a simple and effective tool that has already been used in evaluating the prognosis of diverse cardiovascular diseases, and has demonstrated significant predictive value [26,29]. The COAPT Trial has demonstrated that individuals with heart failure and secondary mitral regurgitation exhibit increased all-cause mortality at 4 years when their GNRI is ≤ 98 [32]. Sun et al. have also shown GNRI is negatively correlated with major adverse cardiovascular events in patients undergoing percutaneous coronary intervention [19]. To our knowledge, this is the first study exploring the relationship between GNRI and outcomes in TBAD patients treated with TEVAR.

The relationships between the nutritional status and TBAD patients' prognosis may be attributed to the following mechanisms: Firstly, metabolism and aging are widely recognized to have a close relationship with nutritional status. Malnutrition is a prevalent metabolic issue among the older population, and preventing malnutrition may reduce the effects of aging and age-related illnesses [33]. Studies have shown that metabolic abnormalities may affect aortic remodeling in TBAD patients, as well as adverse events after TEVAR [34]. Albumin, the critical nutrition element, may influence vascular integrity [35,36],

which is also thought to be a specific inhibitor of endothelial cell apoptosis and protects blood vessels from adverse remodeling [37,38]. In our study, patients in Q1 exhibited higher age and BMI, highlighting the critical role of aging and metabolic factors. However, after adjusting for age and BMI index, we found that the GNRI remained associated with prognosis, suggesting that it may affect outcomes through other mechanisms. Our subsequent analysis revealed a significant increase in the incidence of pericardial effusion in Q1 patients. Prior research has shown that malnutrition increases the risk of pericardial effusion, which was the independent risk factor for poor outcome [39]. Finally, inflammation may also contribute to the malnourished TBAD patients' poor prognosis. Research has demonstrated a significant association between malnutrition and elevated levels of inflammatory markers, which are critical in the development of postoperative complications [40,41]. Albumin exhibits antioxidant and anti-inflammatory properties; therefore, diminished levels of albumin may hinder the body's capacity to regulate inflammatory responses and adversely affect aortic remodeling [42]. In turn, the inflammatory response can inhibit albumin synthesis, thereby exacerbating malnutrition and perpetuating a vicious cycle that accelerates disease progression [43]. Overall, these factors collectively contribute to the poor prognosis of malnourished TBAD patients, and GNRI integrates albumin levels and BMI-related indexes to better reflect patients’ health.

In stratified analysis (Fig. 4), we found the GNRI score exhibited a linear negative relationship with five-year survival rates in the CKD patients. However, in the non-CKD population, the relationship between GNRI and five-year all-cause mortality was not statistically significant, which may be attributed to the potential U-shaped relation. CKD patients normally have poor nutrition status, and high GNRI acts as a protective factor in these populations. Nevertheless, both excessively high and low GNRI levels may lead to adverse outcomes in non-CKD populations. Research has shown that lower GNRI scores are often associated with decreased serum albumin levels, reduced lean body mass, and increased inflammatory markers [44]. Improving nutritional status and enhancing GNRI scores in the CKD population is essential in reducing the risk of adverse outcomes [45]. For the management of CKD patients with low GNRI, protein intake restrictions must be carefully considered [46]. Protein intake can be raised for dialysis patients who do not need preservation of renal function. For patients requiring renal function preservation, intravenous albumin injection and dietary construction adjustments to guarantee sufficient energy intake are practical means to enhance nutritional status [47,48].

As presented in Fig. 3, RCS prognostic analysis reveals a U-shaped curve in the relationship between GNRI and outcomes of TBAD patients. Both extremely low and excessively high GNRI scores are associated with poor prognosis in mid-term prognosis. An abnormally high GNRI score could be attributed to severe obesity, elevated plasma lipid levels, increased systemic inflammation, insulin resistance, and obstructive sleep apnea syndrome [[49], [50], [51]], all of which contribute to unfavorable outcomes in CVD [52].

In summary, GNRI represents the general body metabolism, aging, inflammation as well as the body's resistance to risk factors. Our findings reinforce its predictive value, suggesting that GNRI could be a pivotal clinical tool for risk stratification and personalized care in TBAD patients undergoing TEVAR. By combining serum albumin levels with dynamic weight balance, GNRI enables the early identification of high-risk cohorts—especially those with a GNRI below 98, who are more vulnerable to postoperative complications and mortality. Clinically, this metric facilitates tiered interventions: malnourished patients can benefit from targeted nutritional optimization, such as protocol-driven albumin supplementation (e.g., 0.5 g/kg IV for hypoalbuminemia) and protein regimens tailored to renal function, while overnourished individuals (GNRI > 122) may require calibrated metabolic control through protein restriction and hypocaloric protocols. Postoperatively, serial GNRI monitoring at 24-h intervals can guide adjustments in the recovery trajectory, including modifications to enteral nutrition delivery or the initiation of anti-catabolic therapies. Implementing GNRI-driven pathways would standardize preoperative risk mitigation, enhance intraoperative resilience, and might reduce critical endpoints such as post-TEVAR organ failure, delayed aortic remodeling, and 30-day readmissions. Its integration into clinical workflows offers a unified framework for nutritional stewardship, effectively linking prognostic insight with actionable therapeutic strategies to improve both short-term outcomes and long-term survival in TBAD populations. Compared to other methods, GNRI measurement is simple, rapid, and reproducible, making it highly valuable for preoperative assessment, and postoperative surveillance. Establishing threshold values would further support the development of clinical guidelines, and extensive prospective studies are essential to validate its reliability and reproducibility across diverse patient populations.

## 5 Limitations

Our research possesses several limitations. Firstly, this retrospective observational study from a single center may include selection bias. Multi-central research is needed to amplify data to verify this discovery. Secondly, this study exclusively assessed the baseline GNRI of patients and did not acquire longitudinal data on GNRI variations. Investigating the potential impact of dynamic changes in this indicator on the prognosis of B-type dissection warrants further exploration. Thirdly, the enrolled patients were Chinese, and the metabolic status of different ethnic populations was different, so whether the results can be generalized requires more trials.

## 6 Conclusions

This study indicated GNRI, a common and simple indicator, were independent risk factors of 1-year and 5-year all-cause death for TBAD patients receiving TEVAR. Attention should be directed towards excessively low or high GNRI, and prospective, randomized trials are required to investigate the potential value of nutritional therapies.

## CRediT authorship contribution statement

Kaiwen Zhao, Jinzhu Niu, and Yuzhen He contributed to this study equally. Kaiwen Zhao contributed to study design, data collection, data analysis, and manuscript writing. Jinzhu Niu and Yuzhen contributed to data collection, data analysis, and manuscript writing. Lingxu Kong and Wenyao Zhao contributed to manuscript revises. Qingsheng Lu, Shuangshuang Li and Jian Zhou contributed to study design. All authors read and approved the final manuscript.

## Ethics approval and consent to participate

The requirement for consent forms was waived, and the study protocol was approved by the Clinical Research Ethics Committee of Changhai Hospital, Navy Medical University. The protocol number was CHEC-Y2020-042 and date of this approval was 2020-08-21.

## Funding

The study and collection, analysis, interpretation of data, and preparation of the manuscript were supported by the source of funding as follows: the National Natural Science Foundation of China (82270513), and the Shanghai Municipal Health Commission Research Project [20224Y0351].

## Availability of data and materials

The datasets used in this study are available from the corresponding author on reasonable request.

## Declaration of competing interest

The authors declare that they have no competing interests.
