# Supplementary material for: The role of geriatric nutritional risk index in predicting survival of type B aortic dissection patients after thoracic endovascular aortic repair
Source: J Nutr Health Aging. 2025 May 14;29(7):100572. doi: 10.1016/j.jnha.2025.100572 (PMC12173010; doi:10.1016/j.jnha.2025.100572)
Supplement: Supplementary file 1 [file mmc1.docx]

**Supplementary table 1. Univariate and Multivariate Cox proportional hazard modeling analysis for 1-year all-cause mortality (continuous GNRI).**

| **Variable** | **Univariate analysis** | | |  | **Multivariate analysis** | | |
| --- | --- | --- | --- | --- | --- | --- | --- |
|  | **HR** | **95% CI** | ***P*-value** |  | **HR** | **95% CI** | ***P*-value** |
| Age | 1.02 | (1.00, 1.05) | 0.093 |  | 1.01 | (0.99, 1.04) | 0.333 |
| Male | 8.44 | (1.16, 61.58) | 0.036 |  | 11.21 | (1.51, 83.18) | 0.018 |
| Smoking | 0.77 | (0.40, 1.48) | 0.437 |  |  |  |  |
| SBP at admission | 1.01 | (0.99, 1.02) | 0.213 |  |  |  |  |
| DBP at admission | 1.00 | (0.97, 1.03) | 0.755 |  |  |  |  |
| Hypertension | 1.16 | (0.53, 2.53) | 0.719 |  |  |  |  |
| CAD | 2.49 | (0.97, 6.40) | 0.058 |  | 1.70 | (0.62, 4.66) | 0.305 |
| Diabetes mellitus | 2.46 | (1.18, 5.62) | 0.032 |  | 1.90 | (0.78, 4.60) | 0.157 |
| Stroke | 2.80 | (1.09, 7.20) | 0.033 |  | 1.87 | (0.71, 4.96) | 0.206 |
| COPD | 0.89 | (0.31, 2.52) | 0.826 |  |  |  |  |
| CKD | 3.72 | (1.63, 8.49) | 0.002 |  | 3.09 | (1.29, 7.36) | 0.011 |
| Timing of operation |  |  |  |  |  |  |  |
| Acute | 1 |  |  |  |  |  |  |
| Sub-acute | 0.95 | (0.46, 1.96) | 0.895 |  |  |  |  |
| Chronic | 0.64 | (0.19, 2.14) | 0.469 |  |  |  |  |
| Pericardial effusion | 3.38 | (1.48, 7.71) | 0.004 |  | 3.49 | (1.51, 8.08) | 0.004 |
| Pleural effusion | 1.50 | (0.78, 2.89) | 0.229 |  |  |  |  |
| Branch | 0.58 | (0.20, 1.63) | 0.300 |  |  |  |  |
| Adjunct | 1.50 | (0.69, 3.30) | 0.309 |  |  |  |  |
| Hybrid | 0 | (0.00, Inf) | 0.997 |  |  |  |  |
| GNRI | 1.00 | (0.99, 1.00) | 0.022 |  | 0.97 | (0.94, 1.01) | 0.107 |

GNRI, geriatric nutritional risk index; SBP, systolic blood pressure; DBP, diastolic blood pressure; CAD, coronary artery disease; COPD, chronic obstructive pulmonary disease; CKD, chronic kidney disease; HR, hazard ratio; CI, confidence interval.

**Supplementary table 2. Univariate and Multivariate Cox proportional hazard modeling analysis for 1-year all-cause mortality (GNRI groups).**

| **Variable** | **Univariate analysis** | | |  | **Multivariate analysis** | | |
| --- | --- | --- | --- | --- | --- | --- | --- |
|  | **HR** | **95% CI** | ***P*-value** |  | **HR** | **95% CI** | ***P*-value** |
| Age | 1.02 | (1.00, 1.05) | 0.093 |  | 1.02 | (0.99, 1.04) | 0.182 |
| Male | 8.44 | (1.16, 61.58) | 0.036 |  | 12.83 | (1.71, 96.02) | 0.013 |
| Smoking | 0.77 | (0.40, 1.48) | 0.437 |  |  |  |  |
| SBP at admission | 1.01 | (0.99, 1.02) | 0.213 |  |  |  |  |
| DBP at admission | 1.00 | (0.97, 1.03) | 0.755 |  |  |  |  |
| Hypertension | 1.16 | (0.53, 2.53) | 0.719 |  |  |  |  |
| CAD | 2.49 | (0.97, 6.40) | 0.058 |  | 1.66 | (0.60, 4.53) | 0.326 |
| Diabetes mellitus | 2.46 | (1.18, 5.62) | 0.032 |  | 1.66 | (0.66, 4.15) | 0.280 |
| Stroke | 2.80 | (1.09, 7.20) | 0.033 |  | 1.99 | (0.75, 5.32) | 0.168 |
| COPD | 0.89 | (0.31, 2.52) | 0.826 |  |  |  |  |
| CKD | 3.72 | (1.63, 8.49) | 0.002 |  | 2.90 | (1.19, 7.07) | 0.019 |
| Timing of operation |  |  |  |  |  |  |  |
| Acute | 1 |  |  |  |  |  |  |
| Sub-acute | 0.95 | (0.46, 1.96) | 0.895 |  |  |  |  |
| Chronic | 0.64 | (0.19, 2.14) | 0.469 |  |  |  |  |
| Pericardial effusion | 3.38 | (1.48, 7.71) | 0.004 |  | 3.35 | (1.44, 7.79) | 0.005 |
| Pleural effusion | 1.50 | (0.78, 2.89) | 0.229 |  |  |  |  |
| Branch | 0.58 | (0.20, 1.63) | 0.300 |  |  |  |  |
| Adjunct | 1.50 | (0.69, 3.30) | 0.309 |  |  |  |  |
| Hybrid | 0 | (0.00, Inf) | 0.997 |  |  |  |  |
| GNRI groups |  |  |  |  |  |  |  |
| Quintile 1 | Reference |  |  |  | Reference |  |  |
| Quintile 2 | 0.19 | (0.05, 0.66) | 0.009 |  | 0.22 | (0.06, 0.80) | 0.021 |
| Quintile 3 | 0.55 | (0.24,1.27) | 0.162 |  | 0.59 | (0.24, 1.48) | 0.260 |
| Quintile 4 | 0.26 | (0.08, 0.78) | 0.017 |  | 0.26 | (0.08, 0.81) | 0.020 |
| Quintile 5 | 0.36 | (0.14, 0.94) | 0.038 |  | 0.50 | (0.18, 1.40) | 0.186 |

GNRI, geriatric nutritional risk index; SBP, systolic blood pressure; DBP, diastolic blood pressure; CAD, coronary artery disease; COPD, chronic obstructive pulmonary disease; CKD, chronic kidney disease; HR, hazard ratio; CI, confidence interval.

**Supplementary table 3. Univariate and Multivariate Cox proportional hazard modeling analysis for 5-year all-cause mortality (continuous GNRI).**

| **Variable** | **Univariate analysis** | | |  | **Multivariate analysis** | | |
| --- | --- | --- | --- | --- | --- | --- | --- |
|  | **HR** | **95% CI** | ***P*-value** |  | **HR** | **95% CI** | ***P*-value** |
| Age | 1.06 | (1.04, 1.08) | <0.001 |  | 1.05 | (1.03, 1.07) | <0.001 |
| Male | 1.74 | (0.83, 3.64) | 0.145 |  |  |  |  |
| Smoking | 0.84 | (0.51, 1.37) | 0.487 |  |  |  |  |
| SBP at admission | 1 | (0.99, 1.01) | 0.565 |  |  |  |  |
| DBP at admission | 0.99 | (0.96, 1.01) | 0.284 |  |  |  |  |
| Hypertension | 0.90 | (0.52, 1.57) | 0.717 |  |  |  |  |
| CAD | 1.95 | (0.89, 4.27) | 0.096 |  | 1.09 | (0.49, 2.44) | 0.834 |
| Diabetes mellitus | 2.76 | (1.50, 5.07) | 0.001 |  | 1.76 | (0.92, 3.36) | 0.089 |
| Stroke | 3.81 | (1.93, 7.50) | <0.001 |  | 3.13 | (1.52, 6.44) | 0.002 |
| COPD | 2.18 | (1.20, 3.95) | 0.010 |  | 1.54 | (0.85, 2.82) | 0.157 |
| CKD | 2.52 | (1.25, 5.10) | 0.010 |  | 1.89 | (0.90, 3.97) | 0.093 |
| Timing of operation |  |  |  |  |  |  |  |
| Acute | Reference |  |  |  |  |  |  |
| Sub-acute | 1.09 | (0.64, 1.87) | 0.742 |  |  |  |  |
| Chronic | 0.70 | (0.29, 1.66) | 0.417 |  |  |  |  |
| Pericardial effusion | 3.17 | (1.65, 6.07) | <0.001 |  | 3.02 | (1.56, 5.84) | 0.001 |
| Pleural effusion | 1.25 | (0.75, 2.06) | 0.393 |  |  |  |  |
| Branch | 0.54 | (0.25, 1.19) | 0.127 |  |  |  |  |
| Adjunct | 0.82 | (0.40, 1.65) | 0.573 |  |  |  |  |
| Hybrid | 0 | (0.00, Inf) | 0.995 |  |  |  |  |
| GNRI | 0.99 | (0.99, 1.00) | <0.001 |  | 0.97 | (0.95, 1.00) | 0.027 |

GNRI, geriatric nutritional risk index; SBP, systolic blood pressure; DBP, diastolic blood pressure; CAD, coronary artery disease; COPD, chronic obstructive pulmonary disease; CKD, chronic kidney disease; HR, hazard ratio; CI, confidence interval.

**Supplementary table 4. Univariate and Multivariate Cox proportional hazard modeling analysis for 5-year all-cause mortality (GNRI groups).**

| **Variable** | **Univariate analysis** | | |  | **Multivariate analysis** | | |
| --- | --- | --- | --- | --- | --- | --- | --- |
|  | **HR** | **95% CI** | ***P*-value** |  | **HR** | **95% CI** | ***P*-value** |
| Age | 1.06 | (1.04, 1.08) | <0.001 |  | 1.05 | (1.03, 1.08) | <0.001 |
| Male | 1.74 | (0.83, 3.64) | 0.145 |  |  |  |  |
| Smoking | 0.84 | (0.51, 1.37) | 0.487 |  |  |  |  |
| SBP at admission | 1 | (0.99, 1.01) | 0.565 |  |  |  |  |
| DBP at admission | 0.99 | (0.96, 1.01) | 0.284 |  |  |  |  |
| Hypertension | 0.90 | (0.52, 1.57) | 0.717 |  |  |  |  |
| CAD | 1.95 | (0.89, 4.27) | 0.096 |  | 1.17 | (0.52, 2.63) | 0.710 |
| Diabetes mellitus | 2.76 | (1.50, 5.07) | 0.001 |  | 1.66 | (0.85, 3.21) | 0.136 |
| Stroke | 3.81 | (1.93, 7.50) | <0.001 |  | 3.16 | (1.53, 6.50) | 0.002 |
| COPD | 2.18 | (1.20, 3.95) | 0.010 |  | 1.54 | (0.84, 2.80) | 0.161 |
| CKD | 2.52 | (1.25, 5.10) | 0.010 |  | 1.88 | (0.89, 3.98) | 0.097 |
| Timing of operation |  |  |  |  |  |  |  |
| Acute | Reference |  |  |  |  |  |  |
| Sub-acute | 1.09 | (0.64, 1.87) | 0.742 |  |  |  |  |
| Chronic | 0.70 | (0.29, 1.66) | 0.417 |  |  |  |  |
| Pericardial effusion | 3.17 | (1.65, 6.07) | <0.001 |  | 3.13 | (1.61, 6.09) | <0.001 |
| Pleural effusion | 1.25 | (0.75, 2.06) | 0.393 |  |  |  |  |
| Branch | 0.54 | (0.25, 1.19) | 0.127 |  |  |  |  |
| Adjunct | 0.82 | (0.40, 1.65) | 0.573 |  |  |  |  |
| Hybrid | 0 | (0.00, Inf) | 0.995 |  |  |  |  |
| GNRI groups |  |  |  |  |  |  |  |
| Quintile 1 | Reference |  |  |  | Reference |  |  |
| Quintile 2 | 0.33 | (0.15, 0.71) | 0.005 |  | 0.38 | (0.18, 0.83) | 0.015 |
| Quintile 3 | 0.44 | (0.22, 0.88) | 0.020 |  | 0.46 | (0.22, 0.96) | 0.039 |
| Quintile 4 | 0.38 | (0.18, 0.79) | 0.010 |  | 0.47 | (0.22, 1.00) | 0.051 |
| Quintile 5 | 0.31 | (0.14, 0.67) | 0.003 |  | 0.48 | (0.22, 1.06) | 0.070 |

GNRI, geriatric nutritional risk index; SBP, systolic blood pressure; DBP, diastolic blood pressure; CAD, coronary artery disease; COPD, chronic obstructive pulmonary disease; CKD, chronic kidney disease; HR, hazard ratio; CI, confidence interval.

**Supplementary table 5. Univariate Cox proportional hazard modeling analysis for 1-year and 5-year ARAEs.**

| **Variable** | **1-year ARAEs** | | |  | **5-year ARAEs** | | |
| --- | --- | --- | --- | --- | --- | --- | --- |
|  | **HR** | **95% CI** | ***P*-value** |  | **HR** | **95% CI** | ***P*-value** |
| Age | 1.00 | (0.98, 1.02) | 0.992 |  | 1.00 | (0.99, 1.01) | 0.913 |
| Male | 1.81 | (0.90, 3.62) | 0.096 |  | 1.64 | (0.97, 2.78) | 0.064 |
| Smoking | 0.95 | (0.60, 1.49) | 0.813 |  | 0.97 | (0.67, 1.38) | 0.846 |
| SBP at admission | 0.99 | (0.98, 1.01) | 0.387 |  | 1.00 | (0.99, 1.01) | 0.464 |
| DBP at admission | 1.00 | (0.98, 1.02) | 0.765 |  | 1.00 | (0.98, 1.02) | 0.961 |
| Hypertension | 0.98 | (0.58, 1.64) | 0.935 |  | 1.08 | (0.71, 1.65) | 0.708 |
| CAD | 1.54 | (0.71, 3.35) | 0.276 |  | 1.24 | (0.63, 2.44) | 0.535 |
| Diabetes mellitus | 1.06 | (0.49, 2.30) | 0.891 |  | 0.94 | (0.49, 1.80) | 0.862 |
| Stroke | 1.68 | (0.77, 3.66) | 0.190 |  | 1.83 | (0.99, 3.41) | 0.056 |
| COPD | 0.72 | (0.33, 1.57) | 0.408 |  | 0.99 | (0.57, 1.74) | 0.982 |
| CKD | 1.81 | (0.87, 3.76) | 0.114 |  | 1.28 | (0.65, 2.52) | 0.480 |
| Timing of operation |  |  |  |  |  |  |  |
| Acute | Reference |  |  |  |  |  |  |
| Sub-acute | 1.20 | (0.73, 1.98) | 0.469 |  | 1.31 | (0.89, 1.95) | 0.174 |
| Chronic | 1.43 | (0.73, 2.78) | 0.297 |  | 1.76 | (1.05, 2.95) | 0.031 |
| Pericardial effusion | 2.84 | (1.56, 5.15) | <0.001 |  | 2.44 | (1.46, 4.07) | <0.001 |
| Pleural effusion | 1.55 | (0.99, 2.44) | 0.057 |  | 1.53 | (1.07, 2.19) | 0.020 |
| Branch | 1.62 | (0.97, 2.73) | 0.067 |  | 1.29 | (0.84, 1.99) | 0.248 |
| Adjunct | 1.44 | (0.83, 2.50) | 0.196 |  | 1.17 | (0.74, 1.86) | 0.500 |
| Hybrid | 2.04 | (0.50, 8.29) | 0.321 |  | 1.02 | (0.25, 4.15) | 0.973 |
| Continuous GNRI | 1.00 | (0.98, 1.02) | 0.717 |  | 1.00 | (0.98, 1.01) | 0.788 |
| GNRI groups |  |  |  |  |  |  |  |
| Quintile 1 | Reference |  |  |  | Reference |  |  |
| Quintile 2 | 0.60 | (0.56, 2.28) | 0.218 |  | 0.73  1.17  0.89  0.85 | (0.40, 1.32) | 0.295 |
| Quintile 3 | 1.24 | (0.63, 2.44) | 0.536 |  |  | (0.68, 1.99) | 0.576 |
| Quintile 4 | 0.81 | (0.38, 1.73) | 0.595 |  |  | (0.50, 1.59) | 0.704 |
| Quintile 5 | 1.06 | (0.53, 2.13) | 0.875 |  |  | (0.48, 1.51) | 0.576 |

GNRI, geriatric nutritional risk index; SBP, systolic blood pressure; DBP, diastolic blood pressure; CAD, coronary artery disease; COPD, chronic obstructive pulmonary disease; CKD, chronic kidney disease; HR, hazard ratio; CI, confidence interval; ARAEs, aortic-related adverse events.

**Supplementary table 6. Univariate Cox proportional hazard modeling analysis for 1-year and 5-year MACCEs.**

| **Variable** | **1-year MACCEs** | | |  | **5-year** **MACCEs** | | |
| --- | --- | --- | --- | --- | --- | --- | --- |
|  | **HR** | **95% CI** | ***P*-value** |  | **HR** | **95% CI** | ***P*-value** |
| Age | 1.04 | (1.00, 1.08) | 0.036 |  | 1.03 | (1.00, 1.05) | 0.051 |
| Male | 0 | (0.00, Inf) | 0.997 |  | 2.61 | (0.80, 8.53) | 0.113 |
| Smoking | 0.84 | (0.34, 2.08) | 0.711 |  | 1.06 | (0.53, 2.09) | 0.877 |
| SBP at admission | 0.99 | (0.97, 1.02) | 0.606 |  | 1.00 | (0.99, 1.02) | 0.612 |
| DBP at admission | 0.99 | (0.95, 1.03) | 0.572 |  | 1.01 | (0.98, 1.04) | 0.659 |
| Hypertension | 1.24 | (0.41, 3.73) | 0.703 |  | 1.54 | (0.64, 3.71) | 0.339 |
| CAD | 7.22 | (2.74, 19.00) | <0.001 |  | 5.94 | (2.77, 12.73) | <0.001 |
| Diabetes mellitus | 6.13 | (2.41, 15.58) | <0.001 |  | 5.34 | (2.60, 10.97) | <0.001 |
| Stroke | 3.26 | (0.95, 11.21) | 0.060 |  | 5.66 | (2.44, 13.09) | <0.001 |
| COPD | 0 | (0.00, Inf) | 0.997 |  | 0.22 | (0.03, 1.62) | 0.138 |
| CKD | 5.65 | (2.03, 15.69) | <0.001 |  | 2.73 | (1.06, 7.06) | 0.038 |
| Timing of operation |  |  |  |  |  |  |  |
| Acute | Reference |  |  |  |  |  |  |
| Sub-acute | 1.95 | (0.77, 4.91) | 0.157 |  | 1.15 | (0.56, 2.36) | 0.700 |
| Chronic | 0.58 | (0.07, 4.58) | 0.605 |  | 0.54 | (0.13, 2.33) | 0.412 |
| Pericardial effusion | 3.65 | (1.21, 10.99) | 0.022 |  | 2.46 | (0.95, 6.37) | 0.063 |
| Pleural effusion | 1.09 | (0.43, 2.77) | 0.855 |  | 1.04 | (0.51, 2.10) | 0.913 |
| Branch | 0.85 | (0.25, 2.93) | 0.801 |  | 0.93 | (0.38, 2.24) | 0.864 |
| Adjunct | 2.48 | (0.94, 6.54) | 0.065 |  | 1.35 | (0.59, 3.10) | 0.478 |
| Hybrid | 0 | (0.00, Inf) | 0.997 |  | 0 | (0.00, Inf) | 0.997 |
| GNRI groups | 1.00 | (0.96, 1.04) | 0.982 |  | 1.01 | (0.98, 1.04) | 0.563 |
| Quintile 1 | Reference |  |  |  | Reference |  |  |
| Quintile 2 | 0.69 | (0.19, 2.58) | 0.583 |  | 0.73 | (0.24, 2.16) | 0.564 |
| Quintile 3 | 0.66 | (0.18, 2.46) | 0.534 |  | 0.83 | (0.29, 2.37) | 0.727 |
| Quintile 4 | 0 | (0.00, Inf) | 0.997 |  | 0.49 | (0.14, 1.69) | 0.260 |
| Quintile 5 | 0.99 | (0.30, 3.25) | 0.100 |  | 1.17 | (0.45, 3.08) | 0.746 |

GNRI, geriatric nutritional risk index; SBP, systolic blood pressure; DBP, diastolic blood pressure; CAD, coronary artery disease; COPD, chronic obstructive pulmonary disease; CKD, chronic kidney disease; HR, hazard ratio; CI, confidence interval; MACCEs, major adverse cardiovascular and cerebrovascular events.
